# Detection of ovarian cancer (± neo-adjuvant chemotherapy effects) via ATR-FTIR spectroscopy: comparative analysis of blood and urine biofluids in a large patient cohort

**DOI:** 10.1007/s00216-021-03472-8

**Published:** 2021-07-01

**Authors:** Panagiotis Giamougiannis, Camilo L. M. Morais, Brice Rodriguez, Nicholas J. Wood, Pierre L. Martin-Hirsch, Francis L. Martin

**Affiliations:** 1 Department of Obstetrics and Gynaecology, Lancashire Teaching Hospitals NHS Foundation Trust, Preston, PR2 9HT UK; 2 School of Pharmacy and Biomedical Sciences, University of Central Lancashire, Preston, PR1 2HE UK; 3 Biocel Ltd, Hull, HU10 7TS UK

**Keywords:** Ovarian cancer, Chemotherapy, Biofluids, Liquid biopsies, ATR-FTIR spectroscopy, Spectroscopy

## Abstract

Ovarian cancer remains the most lethal gynaecological malignancy, as its timely detection at early stages remains elusive. Attenuated total reflection Fourier-transform infrared (ATR-FTIR) spectroscopy of biofluids has been previously applied in pilot studies for ovarian cancer diagnosis, with promising results. Herein, these initial findings were further investigated by application of ATR-FTIR spectroscopy in a large patient cohort. Spectra were obtained by measurements of blood plasma and serum, as well as urine, from 116 patients with ovarian cancer and 307 patients with benign gynaecological conditions. A preliminary chemometric analysis revealed significant spectral differences in ovarian cancer patients without previous chemotherapy (*n* = 71) and those who had received neo-adjuvant chemotherapy**—**NACT (*n* = 45), so these groups were compared separately with benign controls. Classification algorithms with blind predictive model validation demonstrated that serum was the best biofluid, achieving 76% sensitivity and 98% specificity for ovarian cancer detection, whereas urine exhibited poor performance. A drop in sensitivities for the NACT ovarian cancer group in plasma and serum indicates the potential of ATR-FTIR spectroscopy to identify chemotherapy-related spectral changes. Comparisons of regression coefficient plots for identification of biomarkers suggest that glycoproteins (such as CA125) are the main classifiers for ovarian cancer detection and responsible for smaller differences in spectra between NACT patients and benign controls. This study confirms the capacity of biofluids’ ATR-FTIR spectroscopy (mainly blood serum) to diagnose ovarian cancer with high accuracy and demonstrates its potential in monitoring response to chemotherapy, which is reported for the first time.

**Graphical abstract:**

**Figure:**
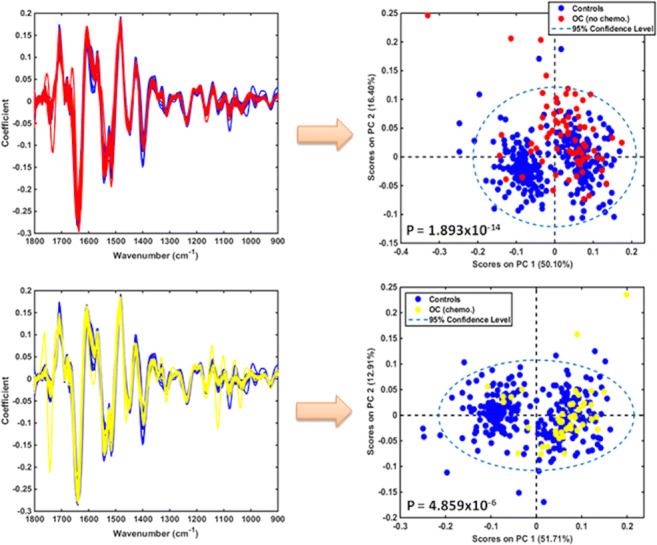
ATR-FTIR spectroscopy of blood serum achieves good segregation of ovarian cancers from benign controls, with attenuation of differences following neo-adjuvant chemotherapy.

**Supplementary Information:**

The online version contains supplementary material available at 10.1007/s00216-021-03472-8.

## Introduction

Ovarian cancer is the 8th most common cancer-related cause of death in women internationally, with incidence and mortality projected to increase markedly in the next 20 years [1, 2]. It also represents the most fatal gynaecological cancer due to non-specific symptoms at its presentation, leading in most cases (> 70%) to being diagnosed in advanced stages of disease [3]. Epithelial ovarian cancer (EOC) represents the most common type (approximately 90% of cases), with histological subclassifications including serous, mucinous, endometrioid, and clear-cell adenocarcinomas and carcinosarcomas. Among these, high-grade serous carcinomas compose the largest group, comprising 70–80% of all EOCs. Other rarer histologies include stromal and germ cell tumours [3, 4]. Current treatment modalities include a combination of surgery and chemotherapy, with the most commonly used chemotherapy agents being platins (either cisplatin or carboplatin) with the addition of a taxane (paclitaxel). In cases where upfront operating is deemed unlikely to achieve complete cytoreduction, chemotherapy is initiated first (in regimes described as neo-adjuvant chemotherapy—NACT) followed by interval debulking surgery (IDS) and subsequent administration of further chemotherapy cycles [4, 5].

In an attempt to increase earlier detection of ovarian cancer, large trials have been conducted, which used a combination of imaging (pelvic ultrasonography) and serum biomarkers [cancer antigen CA125, human epididymis protein 4 (HE4)] to produce screening or diagnostic algorithms [6–9]. However, their outcomes failed to demonstrate robust reduction in mortality rates. Additionally, CA125, which is the most commonly used serum biomarker, lacks adequate sensitivity and specificity as it can be raised in many other non-gynaecological malignancies and non-malignant conditions (such as benign ovarian cysts, uterine fibroids, adenomyosis, and endometriosis) [10, 11]. Particularly in endometriosis, its levels can be significantly elevated in moderate or severe disease [12]. Other tests used in ovarian cancer diagnosis (such as computed tomography, magnetic resonance imaging, tissue biopsies) are expensive, invasive, and time consuming.

The lack of currently available ‘gold standards’ in ovarian cancer detection has led to an urgent need for the development of new accurate, minimally invasive and cost-effective diagnostic tools. One of the promising new modalities is vibrational spectroscopy, which has shown great potential in the classification between normal and pathological biological materials [13]. Attenuated total reflection Fourier-transform infrared (ATR-FTIR) spectroscopy is a frequently used vibrational spectroscopic technique, which has been experimentally applied in human tissues and biofluids [14]. Its main advantage compared to other infrared (IR) techniques is that the evanescent wave produced by the IR source penetrates the sample only by a few microns, rendering it particularly convenient for assessment of biofluids. ATR-FTIR spectra represent a series of wavenumber absorbance intensities produced by molecular motion upon interaction with the IR source [15, 16]. Additionally, the “bio-fingerprint” region in the mid-IR area of the electromagnetic spectrum contains absorbing frequencies for many biomolecules, which can lead to the discovery of new biomarkers [17].

Regarding biological substrates, blood and urine are ideal candidates for cancer screening or diagnosis as they are easy to collect and require minimal (venepuncture) or noninvasive (urine collection) procedures [15]. Blood plasma and serum (which is plasma without clotting factors) contain a multitude of constituents without the presence of erythrocytes, which can interfere with spectra obtained from other important biomolecules, and are considered more suitable biofluids than whole blood for spectroscopic investigations in cancer research [18]. Blood or urine ATR-FTIR spectroscopy has been experimentally applied in several types of cancer including breast [19], brain [20, 21], prostate [22], gastrointestinal [23–25] and endometrial [26, 27]. Its performance has also been explored in ovarian cancer, for investigation of diagnostic accuracy [18, 27] and identification of new biomarkers [27, 28], by studies involving small datasets, with promising results.

For the acquisition of reliable and clinically interpretable results, appropriate chemometric techniques are applied. Vibrational spectroscopic data are inherently multivariate and require complex analytical approaches. Such multivariate methods include principal component (PCA) and linear discriminant analysis (LDA), which can achieve segregation between different classes [29]. The diagnostic potential of spectroscopy can be further enhanced by applying machine learning methods, which utilise a wide range of classification algorithms such as linear discriminant classifier (LDC) and support vector machines (SVMs) [30]. Herein, the potential of ATR-FTIR spectroscopy in ovarian cancer diagnosis was further interrogated, through spectrochemical analyses in blood and urine samples from a large patient cohort. Additionally, as part of recruited ovarian cancer patients had received NACT, chemotherapy effects on acquired spectra were also explored.

## Materials and methods

### Patients and samples

Between April 2018 and November 2019, 423 consecutive patients were recruited (*n* = 116 with ovarian cancer, *n* = 307 with benign gynaecological conditions as controls). The majority of ovarian cancer patients (*n* = 71) had not received chemotherapy whereas the rest (*n* = 45) had received NACT. For the latter group, the median number of NACT cycles was 4 with a median interval of 3 weeks between cycles; 41 patients received a combination of carboplatin and paclitaxel and 4 patients, single-agent carboplatin. Blood samples were collected from all participants and urine from all patients with ovarian cancer. Three patients from the control group did not provide urine. Informed consent was taken from all participants. Samples were collected upon patients’ attendance to Royal Preston Hospital (UK) for surgery and therefore were fasting samples. Serum CA125 levels were measured for all patients at the time of their attendance for surgery and for ovarian cancer patients who received NACT at the time of their disease diagnosis as well. Table 1 contains epidemiological as well as CA125 data for the separate study groups. For NACT ovarian cancer patients, the mean interval between completion of their pre-operative chemotherapy and IDS was 39 days. For each patient, two blood samples were obtained, one in tubes containing EDTA anticoagulant and one in serum gel tubes. Urine was collected following urethral cleansing and sterile urinary catheterisation without the use of lubricant gel. Blood samples were centrifuged at 2200 rpm for 15 min (local protocol), to obtain plasma and serum from EDTA and serum gel tubes respectively. Plasmas and serums were subsequently snap-frozen in liquid nitrogen and stored at − 80 °C. Urines were also stored at − 80 °C without centrifugation and snap-freezing.

**Table 1:** Epidemiological and serum CA125 data for the different study groups. CA125 level was considered elevated if measuring > 35 u/ml

	Mean [range]
Ovarian cancers	
Age	
All patients (*n* = 116)	63 [20–84]
No NACT (*n* = 71)	61 [20–84]
NACT (*n* = 45)	65 [43–83]
ΒΜΙ (kg/m^2^)	
All patients (*n* = 116)	26.7 [16.6–48.6]
No NACT (*n* = 71)	27.4 [18.2–48.6]
NACT (*n* = 45)	25.8 [16.6–36.4]
CA125 (u/ml)	
Non-chemotherapy group (*n* = 71)	590 [5–8366]
Chemotherapy group, before NACT (*n* = 45)	2595 [62–23455]
Chemotherapy group, after NACT (*n* = 45)	135 [8–1104]
Benign controls (n = 307)	
Age	47 [19–89]
BMI (kg/m^2^)	28.5 [17.3–49.8]
CA125 (u/ml)	55 [1–2627]

*NACT* neo-adjuvant chemotherapy

Prior to slide preparation, samples were thawed at room temperature and 60 μl of individual biofluids was pipetted on naked FisherBrand™ and Leica™ glass slides. All slides were allocated a specific serial number for patient confidentiality. Following overnight drying, samples were transferred to the laboratory in wooden slide trays for ATR-FTIR spectroscopic analysis. All slides were stored in a de-humidified glass container to prevent sample condensation and physical damage. The study was granted ethical approval by the East of England - Cambridge Central Research Ethics Committee (archival genito-urinary tissue, blood, urine, saliva and ascitic fluid collection; REC reference: 16/EE/0010; IRAS project ID: 195311).

Identification of pathology for all participants, as well as staging for patients with ovarian cancer, was based on histopathology reports after processing of surgical specimens. All ovarian cancer patients had no other synchronous malignancies. Tables 2 and 3 demonstrate staging data for ovarian cancers and histological diagnoses for the whole cohort, respectively. For most ovarian cancers, the histological subtype was epithelial (with 66% being high-grade serous carcinomas) and only one patient had a germ cell tumour. Staging of ovarian malignancies was conducted according to the International Federation of Gynecology and Obstetrics (FIGO) system [32]. Twenty eight percent of ovarian cancer patients were early stage (FIGO I or II) and all NACT patients had advanced metastatic disease (FIGO III or IV). Further demographic data (including patient comorbidities) are available in non-patient identifiable databases.

**Table 2:** FIGO staging of ovarian cancer patients

	Non-NACT	NACT
ΙΑ	10	–
IC	16	–
ΙΙΑ	5	–
IIB	2	–
ΙΙΙΑ	5	–
ΙΙΙΒ	4	1
IIIC	27	36
IVA	2	5
IVB	–	3

*NACT* neo-adjuvant chemotherapy

**Table 3:** Histopathological data for the entire cohort. Staging of endometriosis patients was based on intra-operative findings according to the American Society of Reproductive Medicine staging system [31]

	No. of patients	
	No NACT	NACT
Ovarian cancers		
High-grade serous	33	43
Low-grade serous	5	–
Primary peritoneal serous	–	1
Mucinous	10	–
Endometrioid	9	–
Clear cell	8	–
Carcinosarcoma	4	1
Anaplastic	1	–
Immature teratoma	1	–
Benign controls		
Ovarian cysts (non-endometriomas)		
Cystadenomas, fibromas, cystadenofibromas	71	
Mature cystic teratomas	13	
Follicular	3	
Haemorrhagic	2	
Struma ovarii	2	
Brenner	1	
Sertoli-Leydig	1	
Indeterminate	2	
Endometriosis (including endometriomas)		
Stage 1	16	
Stage 2	9	
Stage 3	19	
Stage 4	28	
Uterine fibroids and/or adenomyosis	69	
Pelvic inflammatory disease	9	
Endometrial/cervical polyps	8	
Hydrosalpinx/paratubal cysts	7	
Uterine prolapse	3	
Peritoneal leiomyomatosis	1	
Endometrial hyperplasia	1	
Cervical intraepithelial neoplasia (CIN)	1	
Normal (no pathology identified)	41	

*NACT* neo-adjuvant chemotherapy

### Spectral acquisition

ATR-FTIR spectra were obtained via a Bruker TENSOR 27 FTIR spectrometer with Helios ATR attachment, containing a diamond crystal (Bruker Optics Ltd., Coventry, UK) and operated using OPUS 6.5 software. Spectra for each sample were randomly acquired from ten different points. Data acquisition parameters were as follows: 8 cm^−1^ spectral resolution giving 4 cm^−1^ data spacing, 32 scans, 6 mm aperture setting and 2× zero-filling factors. The ATR diamond crystal was cleaned with distilled water and dried with tissue paper between different samples. A background absorption spectrum (for atmospheric correction) was taken prior to each new sample analysis.

### Computational analysis

The chemometric techniques applied in this study have been previously described by our group [33]. Only the spectral fingerprint region was used for data analysis. Spectral pre-processing consisted of the following: Savitzky-Golay (SG) smoothing (window of 7 points, 1st-order polynomial fitting) and 2nd derivative followed by vector normalisation. SG smoothing corrects for random noise, 2nd derivative corrects for baseline distortions and resolves fine spectral structure such as closely aligned peaks (despite potential for reduced signal-to-noise ratio), and vector normalisation corrects for physical differences between samples such as thickness, light scattering and concentrations [34]. In addition, the 10 spectral replicas per sample were averaged in order to work on sample-basis models. Exploratory and discriminant analyses were performed with the pre-processed and mean-centred data. Principal component analysis (PCA) was used for exploratory analysis [35]. With this method, pre-processed spectra are decomposed into a certain number of principal components (PCs), which account for the majority of variance within the dataset. Each PC is composed of scores and loadings; the former is used to detect clustering patterns, relevant to chemical similarities or dissimilarities among samples, and the latter for identification of spectral bands (wavenumbers), which can separate samples from different biological classes, and therefore can be associated with possible spectral biomarkers.

PCA models were built using the PLS Toolbox version 7.9.3 (Eigenvector Research, Inc., USA), and discriminant analysis was performed using the Classification Toolbox for MATLAB [36]. Furthermore, partial least squares discriminant analysis (PLS-DA) was also used as a comparative technique. PLS-DA is a feature extraction and classification algorithm, based on a linear model for which the classification criterion is obtained by PLS [37]. In PLS-DA, a PLS model is applied to pre-processed spectra, reducing the original spectral variables to a few latent variables in an iterative process, where the class labels for each sample are known in the training set. Then, a threshold value that divides the classes’ regions is found [38].

### Statistical analysis

The classification models were evaluated by calculating some figures of merit (accuracy, sensitivity, specificity and F-score) in the test set, composed of 30% of samples selected by using the Morais-Lima-Martin (MLM) algorithm [39]. Training samples, composed of 70% of the dataset, were used for model construction via training and cross-validation [for sample splitting methodology, see Supplementary information (ESM) Table S1]. The accuracy represents the total number of samples correctly classified considering true and false negatives, the sensitivity represents the proportion of positives (i.e. ovarian cancer samples) correctly identified, the specificity represents the proportion of negatives (*i.e.*, benign control samples) correctly identified and the F-score measures the overall model performance considering imbalanced classes [40]. These parameters are calculated as follows:

$$ {\displaystyle \begin{array}{c}\mathrm{Accuracy}\ \left(\%\right)=\left[\left(\mathrm{TP}+\mathrm{TN}\right)/\left(\mathrm{TP}+\mathrm{FP}+\mathrm{TN}+\mathrm{FN}\right)\right]\times 100\\ {}\mathrm{Sensitivity}\ \left(\%\right)=\left[\mathrm{TP}/\left(\mathrm{TP}+\mathrm{FN}\right)\right]\times 100\\ {}\begin{array}{c}\mathrm{Specificity}\ \left(\%\right)=\left[\mathrm{TN}/\left(\mathrm{TN}+\mathrm{FP}\right)\right]\times 100\\ {}\mathrm{F}-\mathrm{score}\ \left(\%\right)=\left(2\times \mathrm{SENS}\times \mathrm{SPEC}\right)/\left(\mathrm{SENS}+\mathrm{SPEC}\right)\end{array}\end{array}} $$

where TP stands for true positives, TN for true negatives, FP for false positives and FN for false negatives. SENS stands for sensitivity and SPEC for specificity.

*P-*values were calculated for two-dimensional PCA score plots using a MANOVA test and for individual wavenumbers based on an ANOVA test. Statistical significance was considered at *P* < 0.05 and statistical high significance at *P* < 0.001.

## Results and discussion

Application of spectroscopy can generate important information about constituents of biological samples. The “fingerprint area” at 1800–900 cm^−1^ in particular provides crucial data, which can lead to characterisation of several key biomolecules [14]. Herein, ATR-FTIR spectroscopy was used to identify the potential of plasma, serum and urine in a large prospective study for the detection of ovarian cancer. The study groups, comprising ovarian cancers with variable histologies and stages of disease, and benign controls with a wide range of gynaecological pathologies, provide a “real-world” clinical setting. Additionally, the consecutive recruitment of participants eliminated the risk of patient selection bias.

**Fig. 1:**
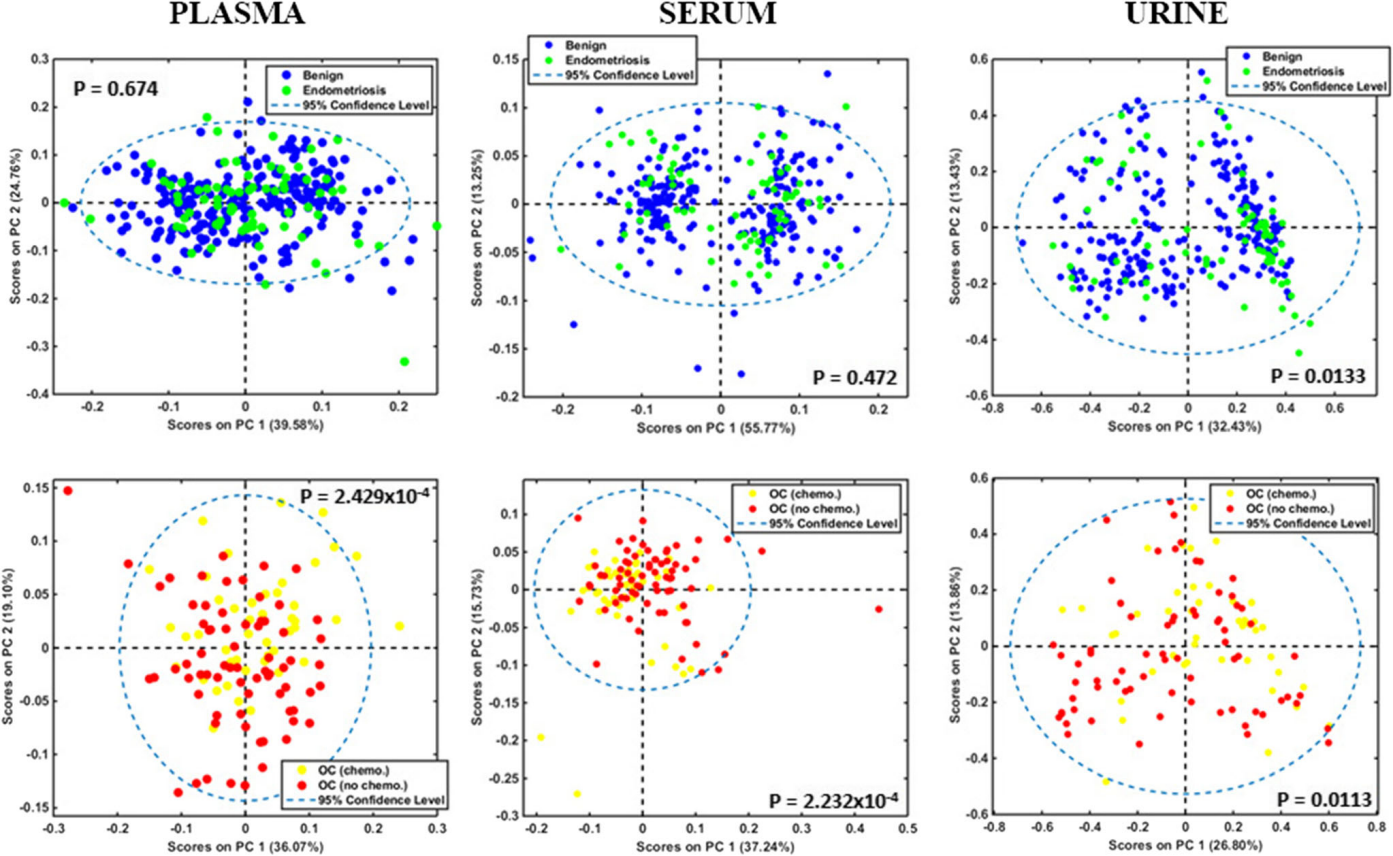
PCA score plots with *P-*values for intra-class comparisons in plasma, serum and urine. Top graphs: non-endometriosis (benign) *versus* endometriosis benign controls. Bottom graphs: non-chemotherapy (OC no chemo) *versus* NACT (OC chemo) ovarian cancer patients. OC, ovarian cancers; chemo, chemotherapy; NACT, neo-adjuvant chemotherapy

An initial exploratory analysis was performed to identify potential significant spectral differences between two subgroups in each of the study’s main patient cohorts (Fig. 1). For controls, this comparison was made between patients with endometriosis (a condition that has a propensity towards ovarian malignancy) [41] and patients with all other benign pathologies. PCA scores identified no difference for blood plasma or serum and a marginal statistically significant difference for urine (*P* = 0.01). Therefore, controls were used as one entity in comparisons. On the other hand, the comparison between ovarian cancer patients without previous chemotherapy and post-NACT reveals high statistically significant differences for blood plasma or serum (*P* ≈ 10^−4^) and marginal statistically significant difference for urine (*P* = 0.01). As this indicated a potential effect of chemotherapy on spectra, these subgroups were compared separately against the whole cohort of controls.

**Fig. 2:**
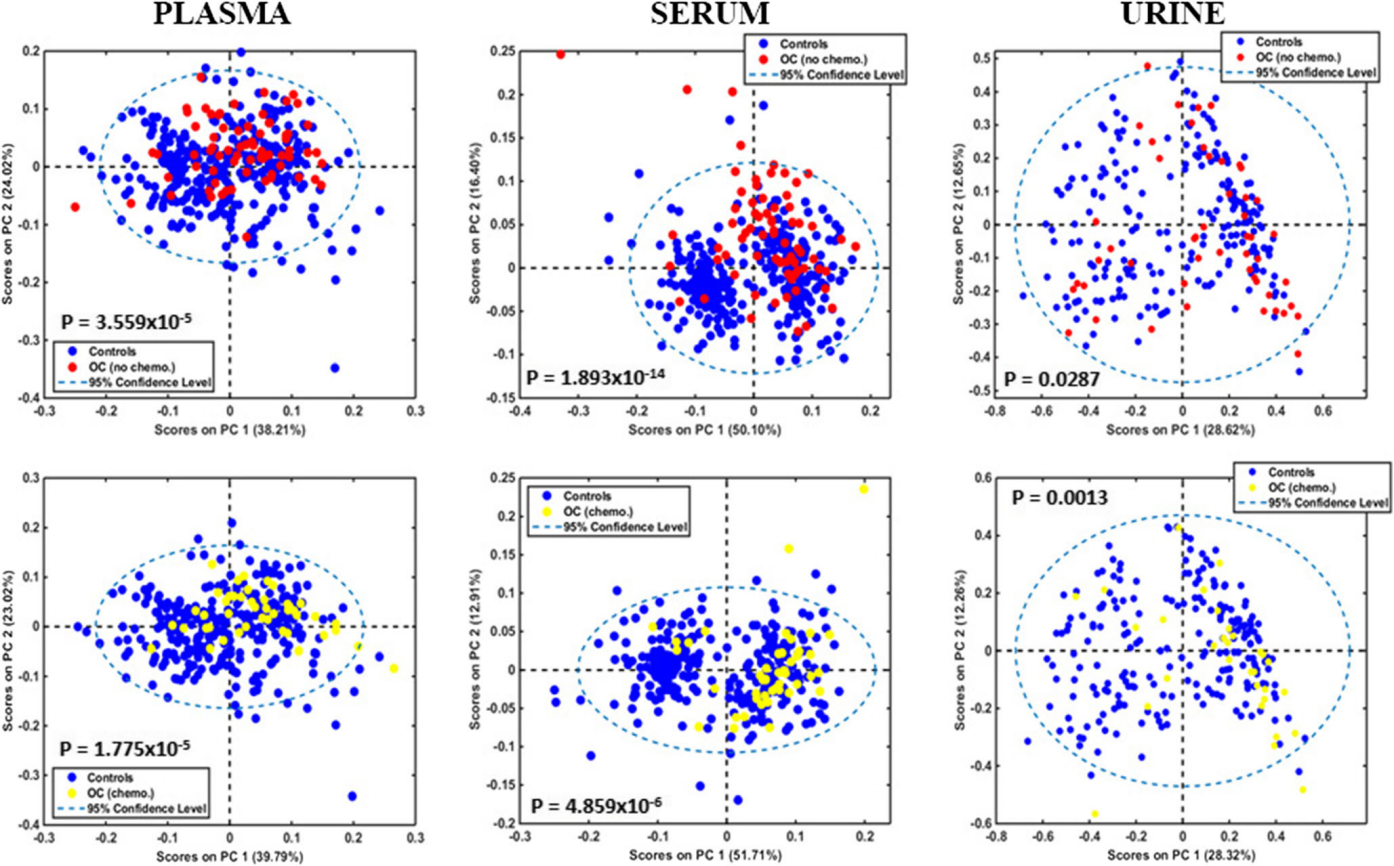
PCA score plots with *P-*values for inter-class comparisons in plasma, serum and urine. Top graphs: non-chemotherapy ovarian cancers (OC no chemo) *versus* all benign controls (controls). Bottom graphs: NACT ovarian cancers (OC chemo) *versus* all benign controls (controls). OC, ovarian cancers; chemo, chemotherapy; NACT, neo-adjuvant chemotherapy

**Fig. 3:**
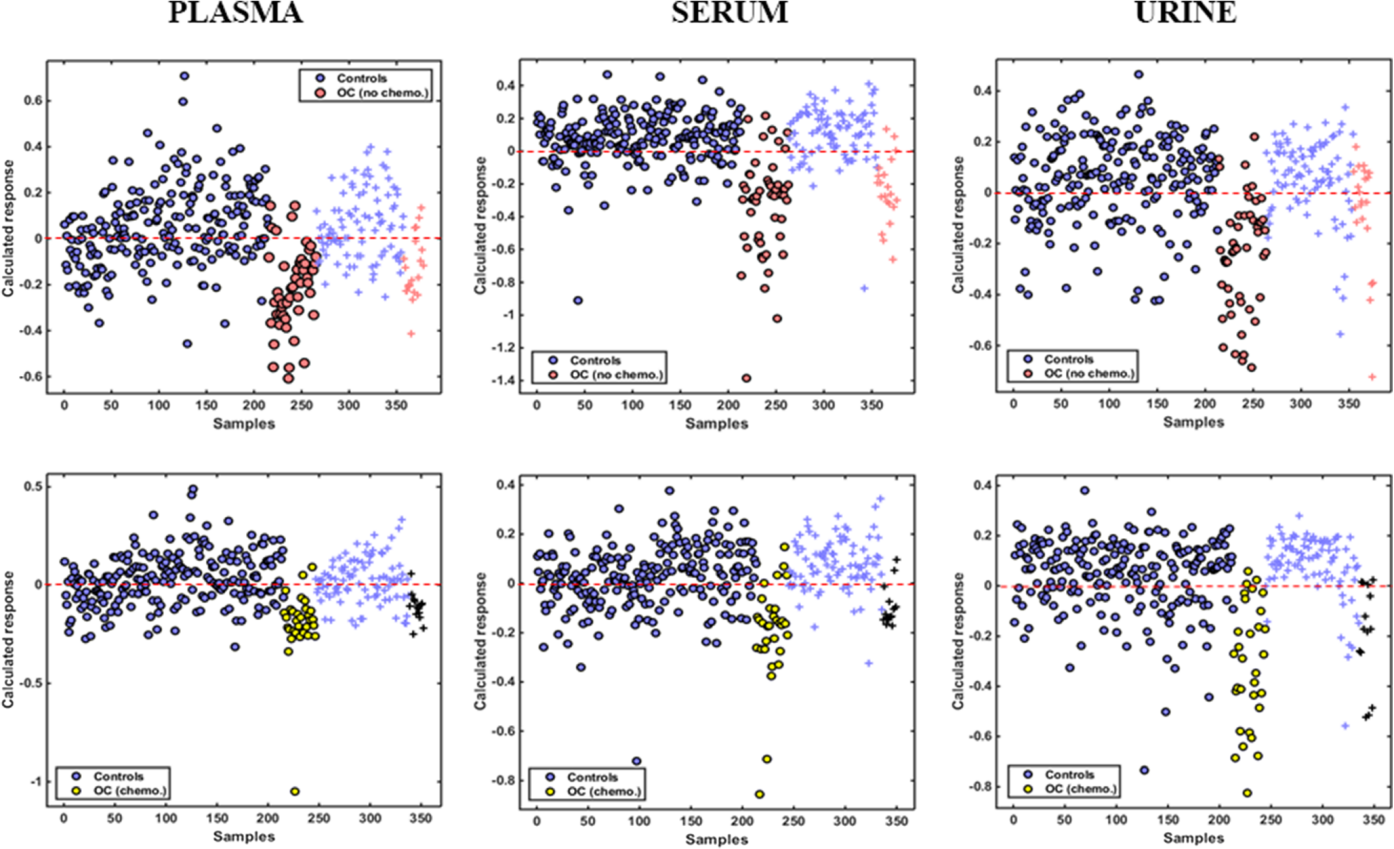
PLS-DA discriminant function plots for plasma, serum and urine. Top graphs: non-chemotherapy ovarian cancers (OC no chemo) *versus* all benign controls (controls). Bottom graphs: NACT ovarian cancers (OC chemo) *versus* all benign controls (controls). OC, ovarian cancers; chemo, chemotherapy; NACT, neo-adjuvant chemotherapy. o = training samples; + = test samples

Exploratory analysis using two-dimensional PCA scores plots suggests that serum is the best for differentiation of both groups of ovarian cancer from benign controls (Fig. 2). PLS-DA was the best discrimination algorithm in all comparisons and for all biofluids, as it consistently achieved the highest F-scores (see ESM Table S2). Figure 3 demonstrates discriminant function plots on training and test samples, obtained with this algorithm for the three biofluids. With regard to blood-derived biofluids, serum achieved the highest sensitivity, specificity and accuracy (76%, 98% and 94% respectively) in the diagnosis of ovarian cancer. Plasma’s statistical metrics were also satisfactory though slightly lower (71%, 84% and 81% respectively). A very interesting finding is the drop in sensitivity for identification of ovarian cancer post-NACT in both biofluids (57% for serum and 64% for plasma), whereas high specificities and accuracies are maintained (85–96%) (Table 4). Similar trends are observed in the other two classification algorithms (PCA-LDA, SVM) used in this study (see ESM Table S2). This finding could suggest a shift towards a more “benign” pattern in spectra obtained from blood biofluids post chemotherapy and is reported for the first time. It is also consistent with the fact that all NACT ovarian cancer patients had chemo-sensitive disease, exhibiting a substantial drop in CA125 at IDS from baseline levels (Table 1), and a reduction in tumour load at their interval computed tomography scan.

**Table 4:** PLS-DA statistical metrics in the classification of the two ovarian cancer groups (non-chemotherapy**—**OC no chemo, NACT**—**OC with chemo) from benign controls for plasma, serum and urine

	Plasma		Serum		Urine	
	OC no chemo	OC with chemo	OC no chemo	OC with chemo	OC no chemo	OC with chemo
Sensitivity	71%	64%	76%	57%	29%	57%
Specificity	84%	88%	98%	96%	87%	92%
Accuracy	81%	85%	94%	91%	76%	88%

*OC* ovarian cancers, *chemo* chemotherapy, *NACT* neo-adjuvant chemotherapy

**Fig. 4:**
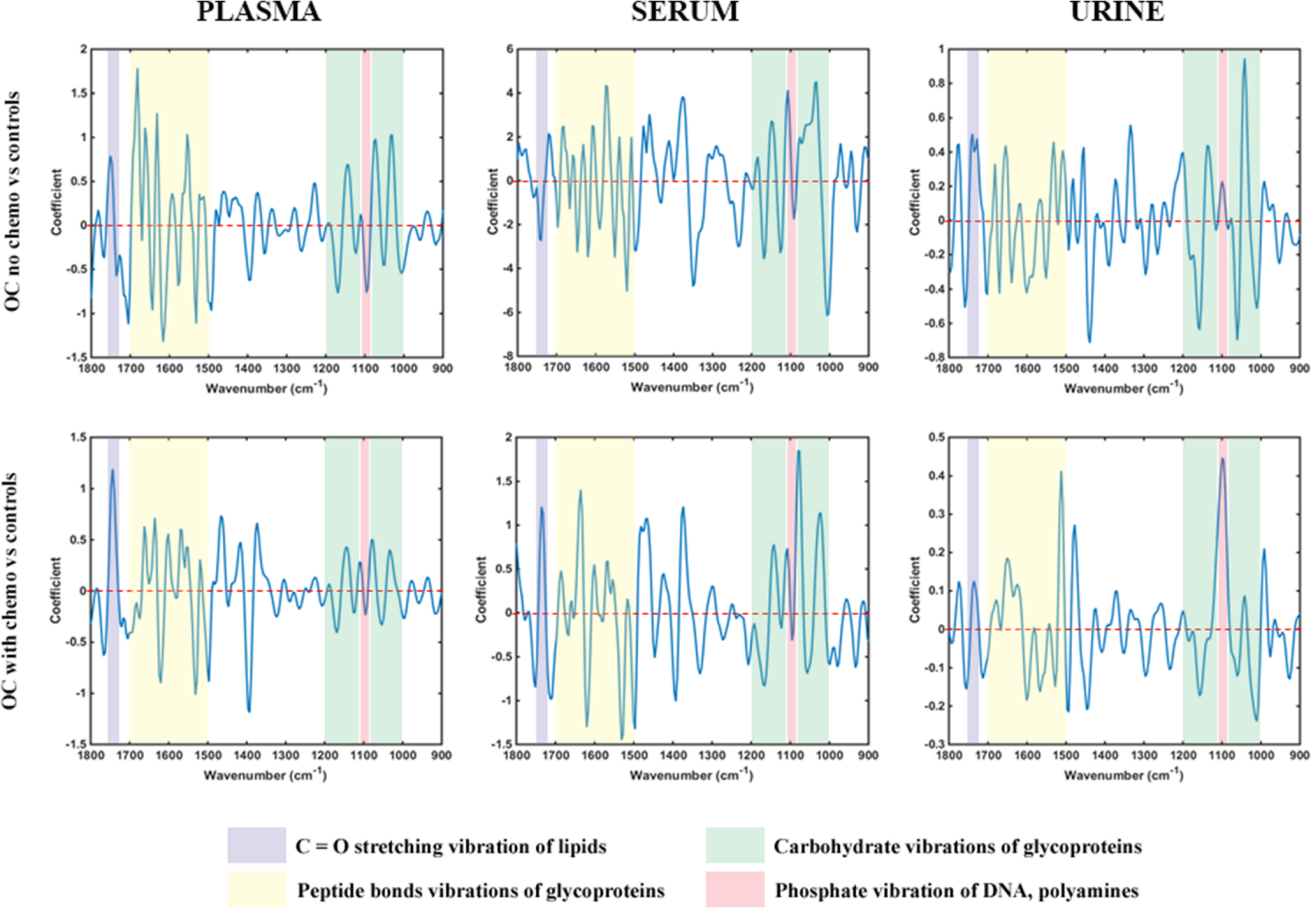
PLS-DA regression coefficient plots for identification of spectral biomarkers in plasma, serum and urine. Key wavenumber regions have been marked with different colours. Top graphs: non-chemotherapy ovarian cancers (OC no chemo) *versus* all benign controls (controls). Bottom graphs: NACT ovarian cancers (OC chemo) *versus* all benign controls (controls). OC, ovarian cancers; chemo, chemotherapy; NACT, neo-adjuvant chemotherapy

Obtained regression coefficient (RC) plots for identification of key biomarkers support the aforementioned conclusion (Fig. 4). In plasma, there were eight peaks with RC > 1 for the non-chemotherapy ovarian cancers but only three for the NACT patients in comparisons with controls. In serum, respective comparisons provided seven peaks with RC > 4 for the former group but none with RC > 2 for the latter. These observations suggest more “subtle” differences between spectra from post-chemotherapy ovarian cancers and benign patients. Additionally, they probably account for the higher sensitivity, specificity and accuracy obtained with serum in classification of ovarian cancers without previous chemotherapy, as well as the bigger drop in sensitivity post-NACT compared to plasma.

Interestingly, in both biofluids, the majority of peaks are observed at 1000–1200 cm^−1^ and 1500–1700 cm^−1^ wavelength regions, which have been assigned to glycoproteins [42, 43]. Spectral absorbances arise from vibrations of carbohydrates’ sugar rings at the former and peptide bonds at the latter [42–44]. Among other glycoproteins that are detected in blood of patients with ovarian cancer and benign gynaecological pathologies (such as HE4, CA15-3, CA72-4), CA125 has by far the highest molecular weight (due to its heavy glycosylation and lengthier protein backbone) and exhibits the widest concentration differences between ovarian malignancy and non-malignant states [45–48]. It is therefore likely that CA125 is the main glycoprotein contributing to absorbances in the two aforementioned wavelength regions. CA125 belongs to the big family of mucins, all being glycoproteins, and CA125 is their largest member [49]. Vibrational bands characteristic for mucins have been described at the spectral region 1040–1120 cm^−1^ [50], at which serum (predominantly) and plasma featured several peaks. Remarkably, absorbance intensities were stronger for the non-chemotherapy ovarian cancers compared to NACT patients in both biofluids, which correlates with the observed magnitude of differences in mean CA125 levels between each of these two groups and controls (Fig. 4, Table 1).

Peaks at RC plots are produced by differences in constituents of biofluids between cases and controls. CA125 has been found to express varied isoforms in ovarian malignancies and benign gynaecological pathologies due to its differential glycosylation in these two entities. In benign conditions, CA125 glycan chains are predicted to contain higher amounts of galactose and N-acetylglucosamine carbohydrates, whereas in ovarian cancer there is a higher content in sialic acid and mannose glycoside residues [51–54]. FTIR spectroscopy has been previously applied for characterisation of carbohydrates [42, 55]. Prominent peaks for sialic acid and mannose have been identified at approximately 1030 cm^−1^ and 1070 cm^−1^, respectively [42], and these peaks had consistently higher absorbance intensities in the ovarian cancer groups compared to controls. On the other hand, peaks that could be attributed to N-acetylglucosamine and galactose (at approximately 1000 cm^−1^ and 1160 cm^−1^, respectively) [42, 55, 56] exhibited consistently higher absorbance in controls (Fig. 4). These observations could suggest a potential for ATR-FTIR spectroscopy to discriminate between benign and malignant CA125 isoforms. Of note, all the aforementioned peaks had lower RCs in the comparisons of NACT patients *versus* controls (Fig. 4), implying a potential for spectroscopic identification of chemotherapy effects in ovarian cancer.

A peak that merits attention is observed at 1740 cm^−1^, assigned to the C=O stretching vibration of lipids [57]. This band demonstrated consistently higher RCs in the NACT group compared to non-chemotherapy ovarian cancer patients in plasma and serum (Fig. 4). Interestingly, use of platinum and taxane chemotherapy agents has been associated with hyperlipidaemia in testicular and breast carcinomas respectively [58, 59], and increased cellular lipid consumption has been associated with chemoresistance in ovarian cancer [60]. Cancer cells utilise high amounts of lipids to meet their energy demands, and increased circulating lipids following effective chemotherapy might reflect a decrease in this metabolic activity with decelerated tumour growth [59, 60]. As all NACT patients in our study had chemo-sensitive disease, their increased blood lipid levels might be an indirect measure of their response to treatment. Although other factors may have contributed to these changes (such as cachexia in ovarian cancer patients without previous treatment and improved appetite leading to higher lipid intake after NACT), spectral changes at 1740 cm^−1^ could exhibit potential for monitoring response to chemotherapy.

Contrary to plasma and serum, PCA scores for urine demonstrated marginal statistically significant differences in the comparisons between ovarian cancer groups and controls (Fig. 2). This was reflected in the poor sensitivity (29%) for diagnosis of ovarian cancer patients without previous chemotherapy obtained with the PLS-DA algorithm. Accuracy was also lower (76%) whereas a fairly high specificity was maintained (87%). A possible reason for these suboptimal results could be the use of un-centrifuged urine, containing contaminants (such as microorganisms and cellular material) that might have obscured signals from important biomolecules. Strikingly though, sensitivity was two times higher for the NACT ovarian cancer group (57%), demonstrating an opposite trend to what was observed in plasma and serum. Specificity and accuracy were also improved (92% and 88% respectively) (Table 4). PCA-LDA and SVM algorithms provided similar trends (see ESM Table S2). Additionally, the majority of peaks at 1000–1200 cm^−1^ and 1500–1700 cm^−1^ wavelength areas had much smaller RCs (< 0.4) and generally exhibited similar amplitudes with peaks in other spectral regions (Fig. 4). This observation suggests that glycoproteins (including CA125) demonstrated much lower absorbance intensities in urine compared to plasma and serum. Indeed, there was only one prominent peak allocated to mucins in the non-chemotherapy ovarian cancer group (at 1040 cm^−1^) [50], which exhibited a substantial drop in NACT patients (from RC 0.9 to 0.1) (Fig. 4). These results are probably due to the markedly low urinary CA125 concentrations, with much smaller mean differences between patients with ovarian cancer and benign gynaecological conditions than the ones present in blood [47, 61].

Taking into consideration the aforementioned findings, it is likely that biomolecules other than glycoproteins contributed to the higher sensitivity obtained in urine for the NACT ovarian cancer patients. The band with the highest RC in this group (and the only one demonstrating a considerable drop in the non-chemotherapy ovarian cancers—from an approximate RC 0.45 to 0.2) was located at 1080–1100 cm^−1^ (Fig. 4). This band has been assigned to phosphate vibrations of DNA but also contains a peak at the IR spectrum of polyamines [57, 62]. Polyamines (spermine, spermidine, putrescine) are low molecular weight molecules that participate in cellular proliferation and DNA synthesis. They are known to interact with DNA bases and phosphate groups, and these adducts produce strong vibrations at the 1080–1100 cm^−1^ spectral domain [63]. Interestingly, the amount of excreted polyamines in urine increases after platinum-based chemotherapy in ovarian cancer, but this effect is mostly observed in patients with chemo-sensitive disease [64]. Therefore, the height of the band at 1080–1100 cm^−1^ may prove useful for monitoring response to chemotherapy, potentially through detection of complexes between polyamines and phosphate on cell-free DNA in urine. Of note, acetylated spermine has been investigated as a possible urinary biomarker for the diagnosis of ovarian cancer [65].

Contrary to what was observed in plasma and serum, absorbance at 1740 cm^−1^ wavelength (produced as previously stated by bonds in lipids) was stronger in the non-chemotherapy ovarian cancer group (RC 0.5) compared to NACT patients (RC 0.1) (Fig. 4). The effect of chemotherapy in urinary lipid concentrations has not been investigated, although a study has reported significantly lower phospholipids in urine of breast cancer patients following surgery (*i.e.*, after reduction or elimination of tumour load) [66]. Therefore, more intense absorbances at 1740 cm^−1^ wavelength in blood biofluids post-NACT, with an opposite trend in urine featuring higher peaks before treatment initiation, might in combination suggest increased chemosensitivity.

An interesting outcome of urine spectroscopy was the presence of a marginal statistically significant difference (*P* = 0.01) between patients with endometriosis and other benign gynaecological abnormalities (Fig. 1). This difference was not present in plasma and serum, suggesting a potential for ATR-FTIR spectroscopy to classify endometriosis in urine samples. Previous studies have revealed some urinary biomarkers (mainly enzymes and peptides) with good discriminatory ability, using methods that included mass spectrometry but not vibrational spectroscopy [67, 68]. However, further chemometric evaluation of this finding was outside the context of this study.

Two previous studies have investigated the performance of biofluids in ovarian cancer diagnosis with ATR-FTIR spectroscopy [18, 27]. In both studies, patients had not received chemotherapy. Gajjar et al. found classification accuracies of 96.67% for plasma and 95% for serum in a cohort comprising 30 ovarian cancer patients and 30 controls with benign gynaecological pathologies. Sensitivities and specificities were not calculated and, although it was stated that 86.7% of ovarian cancer patients had raised CA125 levels, CA125 concentrations were not reported; CA125 levels were not available for the benign control group. In their cohort, 60% of patients had early-stage disease (FIGO I and II) compared to 46.5% in our study for the non-chemotherapy group, with similar distribution of ovarian cancer histological subtypes [18]. Paraskevaidi et al. assessed the performance of urine in a cohort comprising 10 ovarian cancer patients and 10 healthy controls (without stating if benign pathology was present) and found sensitivity, specificity and accuracy of up to 100%, 97.5% and 98.3% respectively. CA125 levels were not available and, although it was reported that all ovarian cancer patients had high-grade disease, staging was not defined [27]. The differences in our outcomes (which were slightly lower for plasma and serum but markedly lower for urine) are probably due to the much bigger size of our cohort, which increased patient heterogeneity, but at the same time provides a more pragmatic estimate of ATR-FTIR spectrochemical performance towards ovarian cancer detection in the general population. This is particularly relevant for the study by Paraskevaidi et al., whose cohort size was approximately 20 times smaller than ours and might have compared advanced-stage ovarian cancer patients to individuals with absent pathology, thus optimising outcomes but at the same time not reflecting a “real-life” situation. The possibility of differences in technical parameters (such as methods of sample collection, preparation and spectroscopic measurements) between the two aforementioned studies and ours was ruled out, as same processes were used. It is also not possible to comment on whether epidemiological factors (such as age, BMI, comorbidities) have affected outcomes, as none of these studies (including ours) performed regression analyses for these variables.

In their study, Paraskevaidi et al. also identified potential diagnostic biomarkers, whereas biomarker assignment for the dataset included in the paper by Gajjar et al. was performed in a separate study by Owens et al. [28]. The latter identified intense absorbances in the ovarian cancer group at 1000–1200 cm^−1^ wavelength area for plasma but not for serum. This is in partial agreement with our findings and reinforces our impression that circulating glycoproteins in blood (predominantly CA125) are strong classifiers of ovarian cancer detected through ATR-FTIR spectroscopy. With regard to urine, there was a single common peak (at 1040 cm^−1^) between our study and the one by Paraskevaidi et al., which as previously stated has been assigned to mucins [50]. Taken together, these observations suggest a consistency of spectroscopic changes that could be attributed to CA125 between different studies, highlighting its importance as a potential biomarker in detection of ovarian cancer through vibrational spectroscopy.

Several systematic reviews have investigated the performance of serum biomarkers in identification of ovarian malignancies. The CA125 assay exhibits 79% sensitivity and 78% specificity, whereas for HE4, sensitivities and specificities range between 74–79% and 87–93%, respectively [69, 70]. In our study, CA125 had similar rates with the ones reported in the literature, featuring 85% sensitivity and 75% specificity at the 35 u/ml cut-off threshold. The combined use of CA125 and HE4 can raise sensitivity up to 87% but specificity does not overcome 82% [70]. Predictive models that use pelvic ultrasonography alone or in combination with CA125 levels have demonstrated sensitivities up to 93% and specificities up to 92% [71]. However, these algorithms rely on the presence of a pelvic mass, which is not always present in ovarian cancer cases. In our study, ATR-FTIR spectroscopy of blood serum achieved 76% sensitivity (overall similar to serum biomarkers) but 98% specificity, which is superior to all the aforementioned modalities, and had excellent diagnostic accuracy (94%). Compared to serum assays, it is more cost-effective and quicker, as it does not require the use of reagents and produces results within minutes (instead of hours or occasionally days). In relation to ultrasonography, it is not operator dependent and can be associated with higher patient acceptance, as it avoids the discomfort and intimacy of transvaginal scanning. Additionally, it does not require the presence of a distinct ovarian tumour, which is a prerequisite in ultrasound-based models, making it applicable to any patient presenting with suspicious symptoms for ovarian cancer. Based on its high specificity, a negative test could reliably exclude the presence of ovarian cancer whereas a positive test would prompt further investigations, thus reducing the amount of unnecessary interventions and patient anxiety.

The main strengths of this study are the size of its cohort (the biggest employed so far in ATR-FTIR spectroscopy of biofluids for ovarian cancer detection) and its prospective design and consecutive recruitment of participants. In this field, the performance of blood-derived biofluids and urine is assessed simultaneously for the first time. It included patients with a wide range of benign gynaecological conditions as well as a variety of ovarian cancer histological subtypes and stages, providing a realistic approach to the encounter of these entities in the general population. It also involves the first attempt to detect chemotherapy-induced spectrochemical changes in biofluids of ovarian cancer patients, and a possible correlation of these alterations with prediction of chemosensitivity. Its main weaknesses are the lack of regression analyses to evaluate the impact of confounding factors, along with the lack of subgroup analyses to estimate performance in early-stage-disease detection, which is the main challenge in the timely diagnosis of ovarian cancer. These parameters would limit direct clinical application of blood serum ATR-FTIR spectroscopy, as a diagnostic tool in ovarian malignancy. Additionally, the assessment of chemotherapy effects was not conducted on a unique ovarian cancer patient group followed up linearly pre- and post-NACT, and as such, the reported outcomes should be regarded as preliminary indirect evidence about the potential of vibrational spectroscopy in investigating treatment response.

In conclusion, our study has shown that ATR-FTIR spectroscopy of blood-derived biofluids (predominantly serum) compares well with currently used diagnostic modalities in ovarian cancer, whereas urine demonstrated poor results. Spectrochemical alterations that can be attributed to circulating CA125 concentration and structural changes can serve as classifiers from benign gynaecological conditions and assessors of chemotherapy effects. Further research, ideally in a single patient cohort with sample collection before and after NACT, is required to validate these results and investigate correlations with tumour resectability at IDS as well as survival outcomes. Finally, future studies should address whether centrifugation can improve the performance of urine in spectroscopic diagnosis of ovarian cancer and determine its potential for classification of endometriosis through ATR-FTIR spectroscopy.

## Supplementary information

ESM 1 (DOCX 1372 kb)
