# Supplementary material for: Detection of ovarian cancer (± neo-adjuvant chemotherapy effects) via ATR-FTIR spectroscopy: comparative analysis of blood and urine biofluids in a large patient cohort
Source: Anal Bioanal Chem. 2021 Jul 1;413(20):5095–107. doi: 10.1007/s00216-021-03472-8 (PMC8405472; doi:10.1007/s00216-021-03472-8)
Supplement: Supplementary file 1 — (DOCX 1372 kb) [file 216_2021_3472_MOESM1_ESM.docx]

**Supplementary Information**

**Detection of ovarian cancer (± neo-adjuvant chemotherapy effects) *via* ATR-FTIR spectroscopy: comparative analysis of blood and urine biofluids in a large patient cohort**

***Journal: Analytical and Bioanalytical Chemistry***

Panagiotis Giamougiannis^1,2^, Camilo L. M. Morais^2^, Brice Rodriguez^1^, Nicholas J. Wood^1^, Pierre L. Martin-Hirsch^1^, Francis L. Martin^3^*

1. Department of Obstetrics and Gynaecology, Lancashire Teaching Hospitals NHS Foundation Trust, Preston PR2 9HT, UK

2. School of Pharmacy and Biomedical Sciences, University of Central Lancashire, Preston PR1 2HE, UK

3. Biocel Ltd, Hull HU10 7TS, UK

***Email:** [flm13@biocel.uk](mailto:flm13@biocel.uk)

**Electronic Supplementary Information:** Contents in the supplementary information file include three figures demonstrating raw, mean raw and pre-processed spectra. Additionally two tables are included, demonstrating sample splitting methodology and statistical metrics for all classification algorithms used in this study.

**Number of Pages = 6**

**Number of Figures = 3**

**Number of Tables = 2**


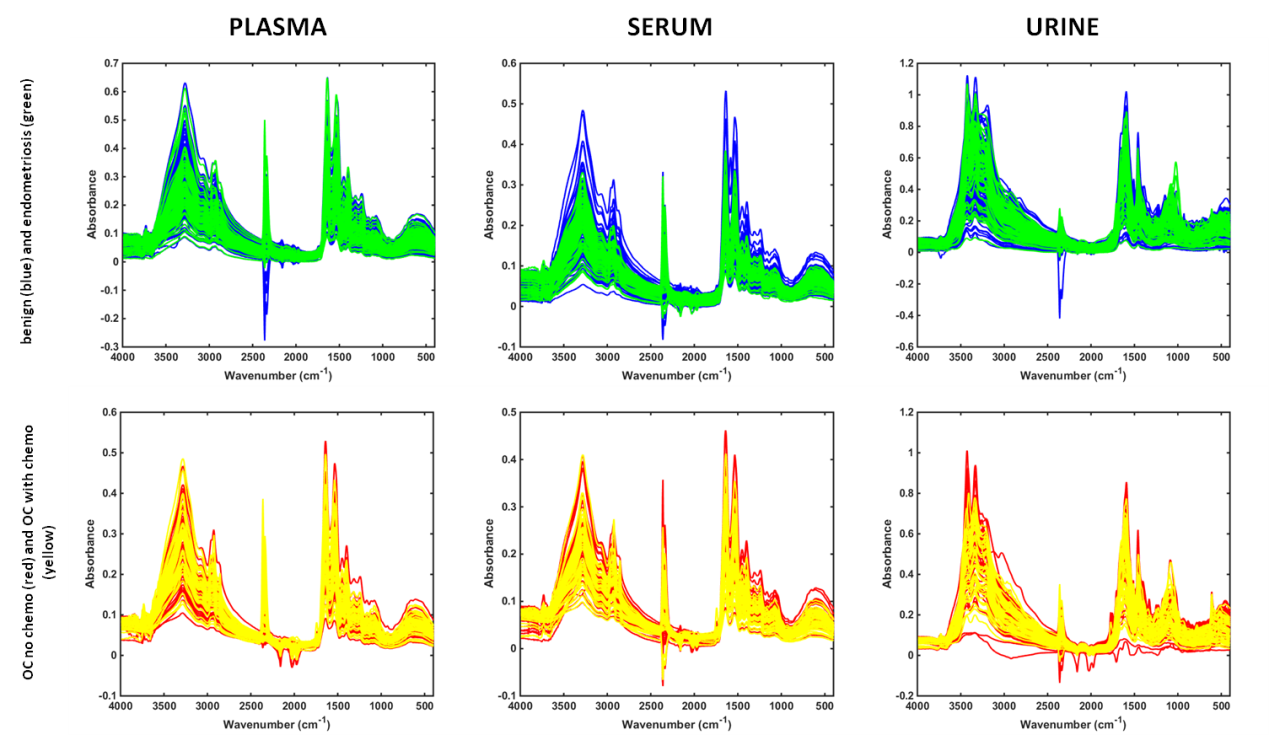


**Fig. S1** Raw spectra of benign control and ovarian cancer classes for plasma, serum and urine. Top graphs: non-endometriosis (benign) and endometriosis controls. Bottom graphs: non-chemotherapy (OC no chemo) and NACT (OC with chemo) ovarian cancers. OC: ovarian cancers, chemo: chemotherapy, NACT: neo-adjuvant chemotherapy


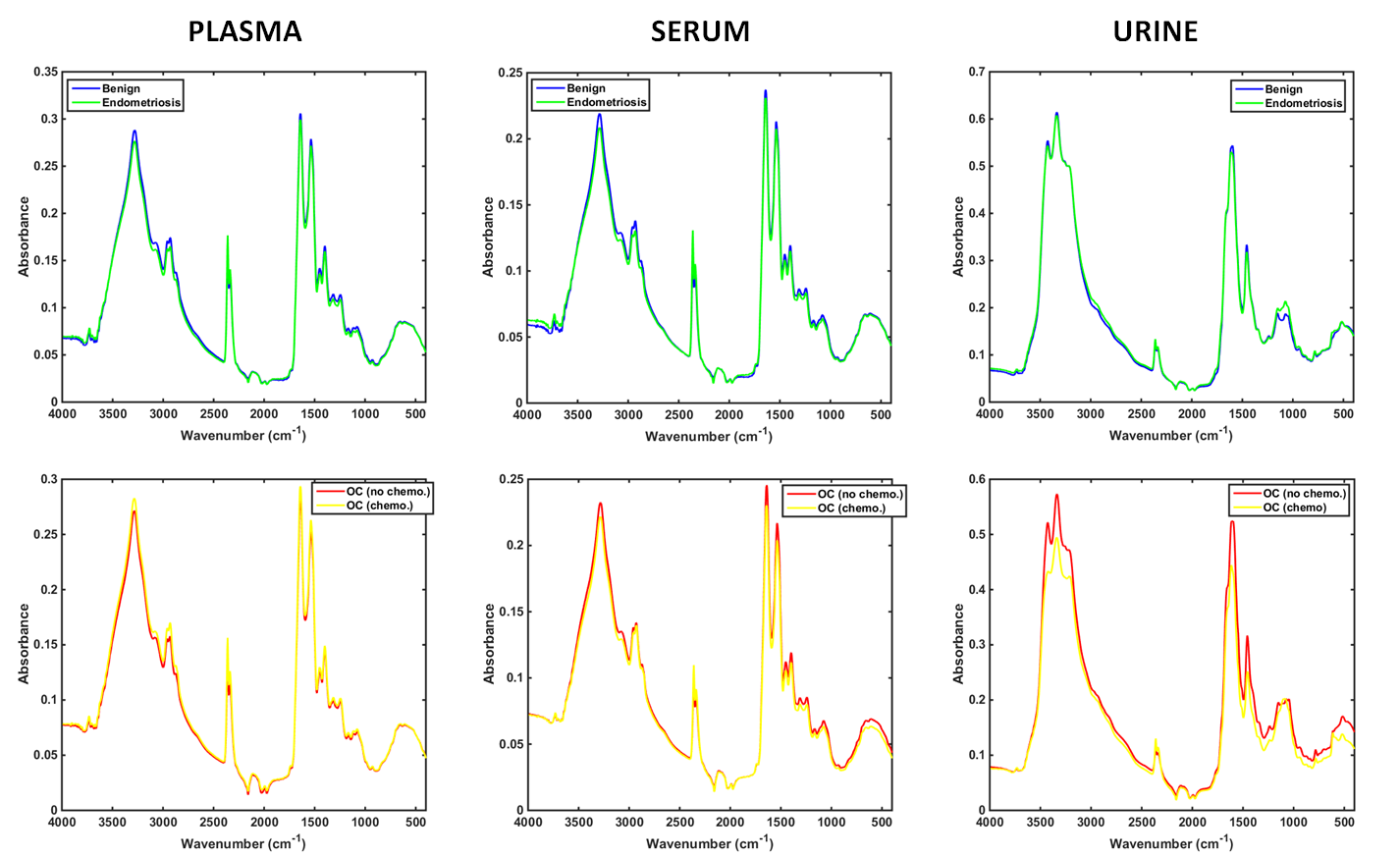


**Fig. S2** Mean raw spectra of benign control and ovarian cancer classes for plasma, serum and urine. Top graphs: non-endometriosis (benign) and endometriosis controls. Bottom graphs: non-chemotherapy (OC no chemo) and NACT (OC chemo) ovarian cancers. OC: ovarian cancers, chemo: chemotherapy, NACT: neo-adjuvant chemotherapy


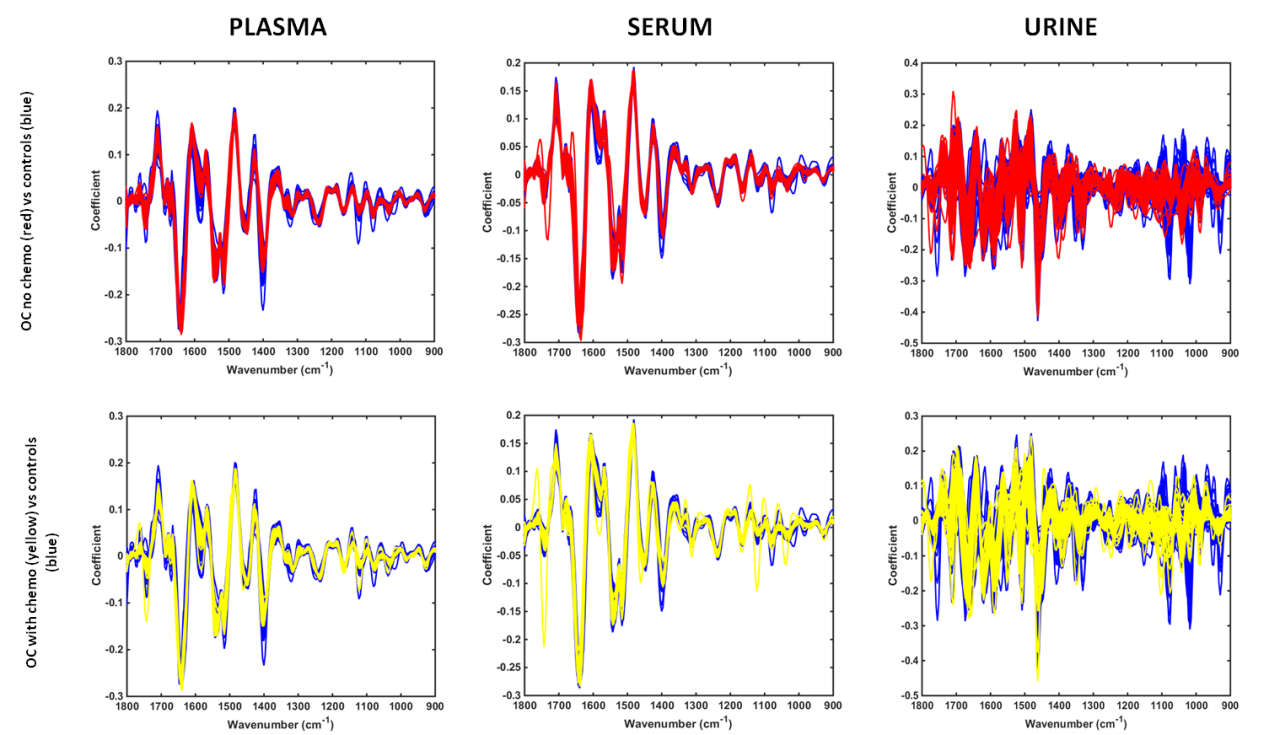


**Fig. S3** Pre-processed spectra of ovarian cancer classes *versus* all benign controls for plasma, serum and urine. Top graphs: non-chemotherapy ovarian cancers (OC no chemo) *versus* controls. Bottom graphs: NACT ovarian cancers (OC with chemo) *versus* controls. OC: ovarian cancers, chemo: chemotherapy, NACT: neo-adjuvant chemotherapy

| **PLASMA** | **SERUM** | **URINE** |
| --- | --- | --- |
| **Classes size:**  Non endometriosis controls = 235  Endometriosis controls = 72  Class 1 (bc + endo) = 307  Class 2 (ovarian cancer no chemo) = 71  Class 2 (ovarian cancer chemo) = 45  **Sample splitting using MLM algorithm**  Training set (70% of samples):  Class 1 & 2 (no chemo) = 265  (215 class 1, 50 class 2)  Class 1 & 2 (chemo) = 246  (215 class 1, 31 class 2)  Test set (30% of samples):  Class 1 & 2 (no chemo) = 113  (92 class 1, 21 class 2)  Class 1 & 2 (chemo) = 106  (92 class 1, 14 class 2) | **Classes size:**  Non endometriosis controls = 235  Endometriosis controls = 72  Class 1 (bc + endo) = 307  Class 2 (ovarian cancer no chemo) = 71  Class 2 (ovarian cancer chemo) = 45  **Sample splitting using MLM algorithm**  Training set (70% of samples):  Class 1 & 2 (no chemo) = 263  (213 class 1, 50 class 2)  Class 1 & 2 (chemo) = 244  (213 class 1, 31 class 2)  Test set (30% of samples):  Class 1 & 2 (no chemo) = 113  (92 class 1, 21 class 2)  Class 1 & 2 (chemo) = 106  (92 class 1, 14 class 2) | **Classes size:**  Non endometriosis controls = 234  Endometriosis controls = 70  Class 1 (bc + endo) = 304  Class 2 (ovarian cancer no chemo) = 71  Class 2 (ovarian cancer chemo) = 45  **Sample splitting using MLM algorithm**  Training set (70% of samples):  Class 1 & 2 (no chemo) = 263  (213 class 1, 50 class 2)  Class 1 & 2 (chemo) = 244  (213 class 1, 31 class 2)  Test set (30% of samples):  Class 1 & 2 (no chemo) = 112  (91 class 1, 21 class 2)  Class 1 & 2 (chemo) = 105  (91 class 1, 14 class 2) |

**Table S1:** Sample splitting methodology used in classification algorithms for plasma, serum and urine. bc: non-endometriosis benign controls, endo: endometriosis, no chemo: non-chemotherapy ovarian cancers, chemo: neo-adjuvant chemotherapy ovarian cancers, MLM: Morais-Lima-Martin algorithm.

| **Biofluid** | **Classification algorithm** | **Accuracy (%)** | | **Sensitivity (%)** | | **Specificity (%)** | | **F-score (%)** | |
| --- | --- | --- | --- | --- | --- | --- | --- | --- | --- |
|  |  | **OC no chemo** | **OC chemo** | **OC no chemo** | **OC chemo** | **OC no chemo** | **OC chemo** | **OC no chemo** | **OC chemo** |
| **Plasma** | | | | | | | | | |
|  | PCA-LDA  (9 PCs no chemo, 3 PCs chemo) | 83 | 86 | 10 | 0 | 100 | 99 | 18 | 0 |
|  | SVM | 88 | 89 | 52 | 36 | 97 | 97 | 68 | 53 |
|  | PLS-DA (4 LVs) | 81 | 85 | 71 | 64 | 84 | 88 | 77 | 74 |
| **Serum** | | | | | | | | | |
|  | PCA-LDA  (6 PCs no chemo, 12 PCs chemo) | 83 | 86 | 19 | 0 | 98 | 99 | 32 | 0 |
|  | SVM | 89 | 86 | 48 | 14 | 99 | 97 | 65 | 25 |
|  | PLS-DA  (10 LVs no chemo, 5 LVs chemo) | 94 | 91 | 76 | 57 | 98 | 96 | 86 | 72 |
| **Urine** | | | | | | | | | |
|  | PCA-LDA  (11 PCs no chemo, 10 PCs chemo) | 80 | 88 | 14 | 21 | 96 | 98 | 24 | 35 |
|  | SVM | 79 | 85 | 10 | 29 | 96 | 93 | 18 | 44 |
|  | PLS-DA  (6 LVs no chemo, 3 LVs chemo) | 76 | 88 | 29 | 57 | 87 | 92 | 43 | 70 |

**Table S2:** Statistical metrics of all algorithms used in classification of the two ovarian cancer groups (non-chemotherapy **-** OC no chemo, NACT **-** OC chemo) from benign controls for plasma, serum and urine. OC: ovarian cancers, chemo: chemotherapy, NACT: neo-adjuvant chemotherapy, PCA-LDA: principal component analysis**-**linear discriminant analysis, SVM: support vector machines, PLS-DA: partial least squares-discriminant analysis, PCs: principal components, LVs: latent variables.
